# State-amplified platform inequality: The economic geography of digital cultural policy in China

**DOI:** 10.1371/journal.pone.0333061

**Published:** 2026-05-18

**Authors:** Li-De Su, Zhi-Hui Pang, Ze-Hao Tan, Zhi-Xiang Wen, Mei Ren, Yang Zhang

**Affiliations:** 1 College of Physical Education, Hunan Normal University, Changsha, China; 2 College of Politics and Administration, Tianjin Normal University, Tianjin, China; 3 Independent person, Windermere, Florida, United States of America; University of Jinan, CHINA

## Abstract

Digital platformization is reshaping not only markets but also the spatial organization of cultural development. While platform concentration has been widely examined in commercial sectors, its territorial consequences within national cultural industries remain underexplored. This study investigates whether China’s 2017 digital cultural policy was associated with widening regional divergence in the cultural economy. We frame this process as state-amplified platform inequality, in which state-led digital policy interacts with platform-based accumulation dynamics to reinforce the advantages of already dominant regions. Using national- and provincial-level data from 2012 to 2023, we combine interrupted time series regression, Bai–Perron structural break tests, and inequality analysis of the radio and television broadcasting sector. The results show that post-2017 growth momentum weakened at the national level and that provincial trajectories became increasingly uneven, with most regions exhibiting negative post-policy slopes. In broadcasting, regional concentration intensified sharply: the Gini coefficient rose from 0.442 to 0.841, the spatial Gini index from 0.352 to 0.689, and the Theil index from 0.439 to 1.839. Structural break tests further identify 2017 as a common breakpoint across key concentration measures. These findings suggest that state-led digital modernization may deepen rather than reduce territorial inequality when implemented in platformized sectors characterized by cumulative advantage. The study contributes a theory-informed account of the economic-geographic and distributive consequences of digital cultural policy and highlights the need to evaluate digital development not only by aggregate growth, but also by its spatial distribution.

## 1. Introduction

Digital transformation has become a central organizing principle of contemporary development policy, and cultural industries have increasingly been drawn into this agenda as sites of both economic upgrading and symbolic production. At the same time, a large body of scholarship has shown that digital platforms tend to concentrate power through network effects, scale economies, data accumulation, and infrastructural control, often producing winner-take-most market structures and highly uneven geographies of value creation [1–3]. Yet these literatures have focused primarily on corporate concentration in market-centered contexts. Much less attention has been paid to how platformized development unfolds when digital transformation is actively organized by the state, and how such state-led digitalization reshapes the territorial distribution of growth within national cultural economies.

This gap is theoretically important because digital cultural policy is often justified in the language of inclusion, modernization, innovation, and national competitiveness [4,5]. Such policies are expected to broaden participation, enhance industrial capacity, and diffuse the benefits of digital development across regions. However, these ambitions may be undermined when policy implementation interacts with platform logics that reward scale, visibility, interoperability, and network centrality. Under those conditions, territories that already possess stronger infrastructures, institutional capacity, political prominence, or platform-linked industrial bases may be better positioned to absorb policy benefits than lagging regions. The key theoretical puzzle, therefore, is not simply whether digital policy has effects, but why state-led digital modernization may intensify territorial concentration rather than diffuse opportunity.

China provides a revealing setting in which to examine this question. Its digital economy has developed through a hybrid configuration in which platform expansion, state coordination, industrial policy, and uneven regional development have advanced simultaneously. In 2017, the Ministry of Culture issued the Guiding Opinions on Promoting the Innovative Development of the Digital Culture Industry, a national policy document that framed digital culture as a priority area for new supply, new consumption, industrial upgrading, and innovation ecosystem building. Official policy interpretation described the document as the first top-level design at the ministerial level specifically devoted to the digital culture industry, linking it to the broader “13th Five-Year” strategic agenda and laying out priorities for key fields, innovation ecosystems, and policy support [6]. This intervention was therefore institutionally significant, but its territorial consequences were unlikely to be uniform because implementation in China is commonly mediated by uneven local state capacity and differential access to resources, administrative leverage, and industrial foundations [7,8].

Existing scholarship does not yet provide a sufficiently integrated explanation for this kind of policy environment. Research on platform capitalism has explained how digital intermediaries consolidate economic and infrastructural power, but often treats the state as a regulator, background condition, or ex post corrective rather than as a constitutive force in the making of digital markets [9,10]. Research on digital governance in China, by contrast, has highlighted state coordination, infrastructural power, and forms of digital leadership, but has said less about how platform accumulation logics and territorial inequality may be mutually reinforced under digital modernization [11]. Studies of regional inequality and uneven development offer valuable insight into spatial divergence, yet they do not always specify how platformization and policy design operate together within cultural industries, where economic value and symbolic influence are deeply intertwined [12].

To address this gap, this study develops the concept of state-amplified platform inequality. We define it as a process in which state-led digital policy interacts with platform-based accumulation dynamics to intensify territorial disparities by disproportionately channeling infrastructure, institutional support, visibility, and market access toward already advantaged regions. The concept shifts the analysis of platform inequality away from a purely market-centered explanation and toward a hybrid political-economic account in which territorial concentration is co-produced by digital platform logics and selective modernization strategies. Rather than asking whether China’s digital cultural policy transformed all dimensions of cultural life, we adopt a more bounded and empirical focus on its economic-geographic and distributive consequences, especially regional divergence in cultural-industrial growth and concentration.

Empirically, we examine whether China’s 2017 digital cultural policy was associated with more uneven provincial trajectories in the cultural economy and whether broadcasting, as a strategically revealing sector, displayed intensified concentration after the policy. Broadcasting is especially important in this context because it occupies a hybrid institutional position at the intersection of state regulation, content distribution, infrastructural scale, and symbolic power. It is therefore not treated here as a proxy for the entirety of digital culture, but as a sectoral case through which the distributive consequences of state-led platformization can be observed particularly clearly.

This study makes two theoretical contributions. First, it develops the concept of state-amplified platform inequality as a framework for explaining why state-led digital modernization may deepen rather than reduce territorial inequality. Second, it provides province-level evidence that a major national digital cultural policy was associated with uneven post-policy trajectories in China’s cultural economy and with intensified concentration in broadcasting as a strategically revealing sector.

## 2. Literature review

### 2.1 Platform capitalism and the spatial concentration of digital accumulation

The literature on platform capitalism has established that digital platforms are not merely neutral intermediaries but infrastructures of extraction, coordination, and market organization [13,14]. Their economic power stems from the capacity to aggregate users, capture data, orchestrate interactions, and convert scale into self-reinforcing advantages. Network externalities, data feedback loops, and the low marginal costs of digital replication allow successful platforms to expand rapidly, extend into adjacent sectors, and consolidate gatekeeping power over access to markets, audiences, and information [15–18]. For this reason, platformization is widely associated with concentration rather than dispersion.

Importantly, this concentration is also spatial. Platform expansion tends to cluster high-value functions such as headquarters, finance, engineering, content moderation, and advertising coordination in a limited number of privileged urban and regional nodes [1,19]. Even where platforms serve national or transnational markets, their control functions and value capture mechanisms remain territorially uneven. Recent work has therefore argued that platform capitalism must be understood geographically, not only sectorally, because its infrastructures generate highly selective landscapes of accumulation, dependence, and centrality [20,21]. The spatiality of platform power is particularly consequential in sectors linked to media and culture, where digital intermediation structures not only markets but also systems of visibility and symbolic circulation [22].

Yet this literature has two limitations for the present study. First, much of it is built around market-centered cases in which corporate strategy is analytically primary and the state appears mainly as regulator, competition authority, or legal backdrop. Second, although it explains why concentration occurs, it says less about how national policy can selectively accelerate that concentration across territories. In other words, platform capitalism scholarship clarifies why digital intermediaries tend toward concentration, but it only partially explains how those dynamics are shaped when the state itself actively organizes the developmental trajectory of digital industries.

### 2.2 State-led digitalization, developmental governance, and uneven territorial implementation

A different body of work emphasizes the centrality of the state in shaping digital development. In the Chinese context especially, digital transformation has unfolded through a dense interplay of industrial policy, infrastructural expansion, political supervision, and local implementation. Research on Chinese digital governance has shown that the state does not simply react to digital markets after they emerge; it often participates in defining strategic sectors, building regulatory frameworks, steering investment priorities, and aligning technological development with broader political and developmental objectives [23–25]. In such settings, digitalization is not just a technological trend but a state-mediated project of modernization.

However, state-led development does not occur across homogeneous territory. A long tradition of scholarship on Chinese governance and implementation has shown that national policies are filtered through uneven local capacity, fiscal conditions, administrative incentives, and regionally differentiated development strategies [26–28]. This means that even nationally framed projects may produce selective territorial outcomes in practice. Regions with stronger institutional capacity, more developed industrial ecosystems, greater political centrality, or preexisting infrastructural advantages are often better positioned to translate policy signals into durable growth than regions with weaker starting conditions. In this sense, the territorial consequences of policy are shaped not only by formal design but also by the unequal geography of implementation.

Still, the state-led digitalization literature also remains incomplete for our purposes. While it helps explain how policy steering, coordination, and implementation matter, it does not always specify the platform-specific mechanisms through which selective territorial gains may be magnified. Platformized sectors reward scale, interoperability, centrality, and visibility in ways that can transform modest prior advantages into self-reinforcing dominance. Thus, to understand the regional consequences of digital cultural policy, it is not enough to know that the state matters; we must also understand how state action interacts with platform logics that systematically privilege already advantaged nodes.

### 2.3 Cultural industries, symbolic power, and the strategic relevance of broadcasting

Cultural industries occupy a particularly important position in this debate because they are simultaneously economic sectors and infrastructures of symbolic production. Their outputs carry commercial value, but they also shape representation, legitimacy, public communication, and the circulation of cultural meaning. This dual character makes them especially sensitive to the interaction between state priorities and digital platforms. In market terms, digital cultural production is increasingly mediated by scalable platforms that structure distribution, recommendation, and audience access. In political and symbolic terms, culture remains deeply entangled with questions of governance, legitimacy, and national representation.

Within this broader field, broadcasting is a strategically revealing sector. In China, broadcasting has long been tied to state oversight and institutional hierarchy [29], yet it has also been transformed by digitization, platform-based dissemination, and the integration of legacy media into broader digital ecosystems [30]. This dual embeddedness makes broadcasting analytically valuable for studying territorial concentration under state-led platformization. It is a sector in which regulatory authority, infrastructural reach, cultural legitimacy, and digital scalability intersect more visibly than in many other domains. For that reason, broadcasting should not be read as a proxy for all digital culture, but as a sectoral case through which the distributive and territorial consequences of digital cultural policy can be observed with unusual clarity. Research on Chinese media has long noted that media commercialization and digital transformation have not displaced state power so much as reconfigured it through new institutional and technological arrangements [31–34]. The present study extends that insight by asking whether these transformations are also associated with a more unequal regional economic geography. This strategic relevance motivates the later use of broadcasting as the sectoral site for structural-break and interprovincial inequality analyses.

### 2.4 Toward a theory of state-amplified platform inequality

Building on the foregoing literatures, we propose the concept of state-amplified platform inequality to explain a specific mechanism of uneven development in state-led digital economies. The concept refers to a process in which public policy does not counteract platform concentration but instead interacts with it in ways that intensify territorial disparities. When digital policy channels recognition, infrastructure, finance, regulatory support, and developmental legitimacy toward designated sectors and preferred nodes, territories that already possess stronger institutional and infrastructural foundations are better positioned to convert those advantages into scalable platform-linked growth. Platformization then magnifies those gains because success in digital markets is strongly cumulative: greater visibility attracts more users, more users generate more data, more data support further optimization, and optimization reinforces market centrality.

The key theoretical claim is therefore relational. Territorial inequality in digital cultural development should not be understood as the simple result of spontaneous market concentration, nor as a generic byproduct of state favoritism. Rather, it emerges from the interaction between platform accumulation logics and selective modernization strategies. The state identifies priorities, certifies developmental pathways, and allocates support in ways that are territorially differentiated. Platforms transform those differentials into cumulative advantages through scale, intermediation, and concentration. The result is a hybrid form of uneven development in which digital modernization may increase aggregate capacity while also deepening spatial asymmetry.

This framework differs from adjacent theories in several ways [35]. It extends platform capitalism scholarship by treating the state as constitutive of territorial platformization rather than as an external regulator. It extends research on state-led digitalization by specifying the platform-specific mechanisms through which selective support can become cumulative regional advantage. It also differs from generic digital divide accounts, which often focus on access differentials, by emphasizing how policy design and platform infrastructures co-produce territorial concentration in economic value and institutional visibility. Finally, it extends regional inequality research by locating spatial divergence within a specific political-economic configuration: the convergence of platformized accumulation and state-directed digital modernization.

### 2.5 Policy background and intervention logic

The policy intervention examined in this study is the 2017 Guiding Opinions on Promoting the Innovative Development of the Digital Culture Industry, issued by the Ministry of Culture of the People’s Republic of China [6]. The document is widely treated in official policy discourse as the first ministerial-level top-level design specifically dedicated to the digital culture industry, and it framed digital culture as a priority area for industrial upgrading, technological integration, platform development, and new consumption formation. Rather than representing a minor administrative adjustment, the policy articulated a national development agenda that sought to expand digital cultural production, support innovation-oriented enterprises, foster new forms of online cultural consumption, and promote the construction of digitally enabled cultural industry systems.

The analytical importance of the 2017 intervention lies not only in the existence of a dated policy document, but in the institutional mechanisms through which it could plausibly affect provincial outcomes. By elevating digital culture within the broader framework of national modernization and industrial transformation, the policy provided political recognition, developmental legitimacy, and implementation direction for local governments and firms. In practice, such guidance could influence provincial cultural economies through several channels, including infrastructure investment, preferential support for digital cultural enterprises, regional industrial base construction, innovation incentives, and closer alignment between local planning and national strategic priorities. Because these implementation capacities and preexisting industrial conditions were unlikely to be evenly distributed across provinces, a theoretically important possibility is that the policy may have reinforced rather than reduced territorial divergence. For that reason, 2017 is treated in this study as an institutionally meaningful intervention point whose post-policy consequences can be examined through segmented trend analysis.

### 2.6 Empirical expectations

The framework developed above generates two empirical expectations for the present study. First, if state-led digital policy interacts with platform dynamics in the manner proposed here, post-policy development should not necessarily appear as broad-based territorial diffusion. Instead, one would expect a more uneven pattern in which some provinces consolidate advantage while others stagnate or decline relative to pre-policy trajectories. This logic suggests that the distributive consequences of digital cultural policy may be detectable not only in aggregate national trends but also in widening provincial heterogeneity.

Second, strategically important sectors situated at the intersection of state coordination, symbolic authority, and digital dissemination should show intensified concentration after the policy intervention. Broadcasting is especially relevant because its hybrid institutional position makes it highly responsive both to state developmental priorities and to platformized forms of scaling and distribution. An increase in interprovincial inequality within this sector would therefore provide strong evidence consistent with the theory.

Taken together, these expectations imply a broader proposition: state-led digital modernization may generate aggregate upgrading while simultaneously deepening territorial divergence. The central question of the empirical analysis is therefore whether China’s 2017 digital cultural policy was associated with such an outcome in the cultural economy and, more specifically, whether broadcasting exhibited a post-policy pattern of intensified regional concentration consistent with state-amplified platform inequality.

## 3. Methods

### 3.1 Research design

This study adopted a longitudinal quasi-experimental design to examine whether China’s 2017 digital cultural policy was associated with changes in the economic geography of the cultural industry. The empirical objective was not to evaluate the total social or cultural effects of the policy, but to assess its economic-geographic and distributive consequences, particularly changes in growth trajectories across provinces and changes in regional concentration within a strategically important cultural sector. This bounded scope aligns the empirical design with the study’s theoretical interest in state-amplified platform inequality.

**Fig 1:**
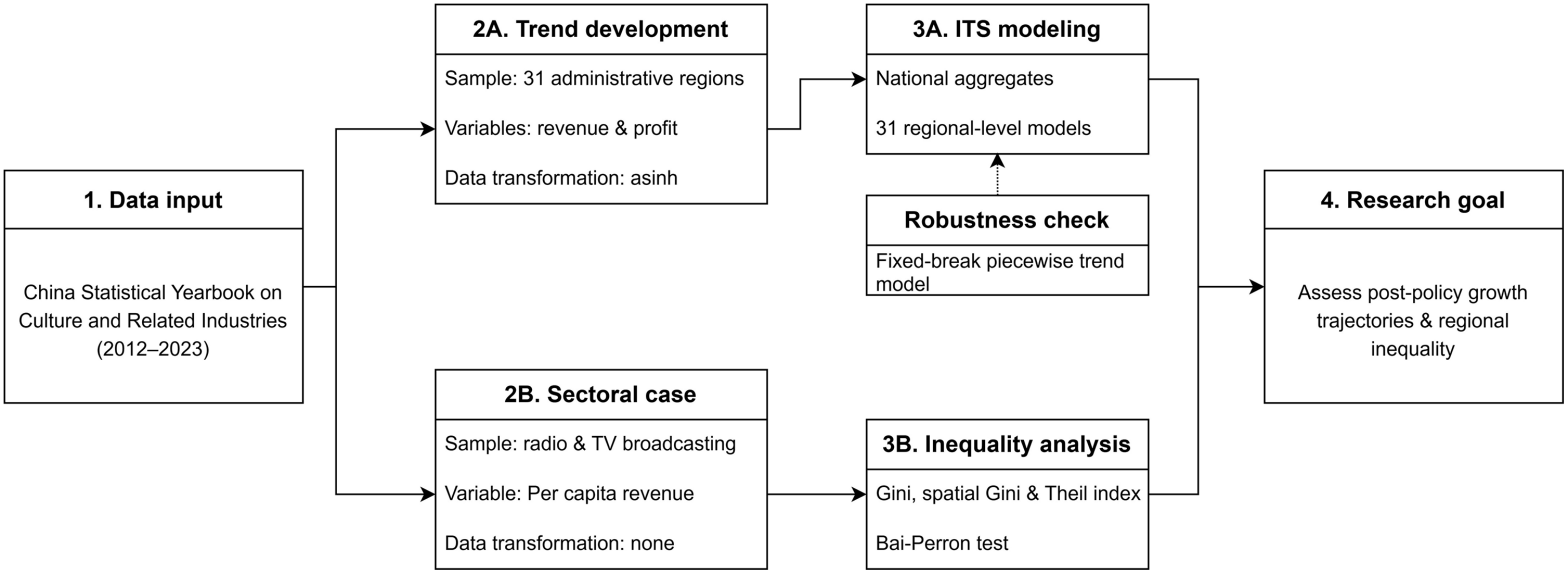
Research workflow of the empirical analysis.

To address this question, the analysis combined interrupted time series (ITS) modeling with structural break detection and interprovincial inequality analysis. The unit of analysis differed across the three empirical components. In the national ITS models, the unit of analysis was the annual national aggregate for each revenue or profit outcome. In the provincial ITS models, the unit of analysis was the annual province-level series for each of China’s 31 administrative regions. In the broadcasting-sector concentration analysis, the unit of analysis was the annual cross-provincial distribution of per capita broadcasting revenue. This multi-level design allowed the study to examine aggregate policy-associated change, regional heterogeneity in post-policy trajectories, and the evolution of territorial concentration within a strategically important sector. Fig 1 summarizes this workflow.

### 3.2 Broadcasting as a strategic sectoral case

The radio and television broadcasting sector was selected as a strategic sectoral case because it occupies a distinctive position in China’s cultural economy. Broadcasting combines strong state oversight, broad infrastructural reach, and continued importance in content distribution, making it especially useful for observing whether digital cultural policy was associated with intensifying regional concentration. Unlike some other cultural sectors, broadcasting remains closely tied to institutional authority and symbolic coordination while also becoming increasingly embedded in digital dissemination and platform-centered media ecologies. For this reason, changes in the spatial distribution of broadcasting revenue provide a particularly revealing window into the distributive consequences of state-led digitalization. Empirically, the broadcasting sector was examined through two complementary strategies: temporal structural-break analysis to identify major shifts in development trajectory, and annual cross-provincial inequality analysis to assess whether territorial concentration intensified over time. Broadcasting is therefore used not as a proxy for all cultural outcomes, but as a strategically important sector in which uneven territorial concentration can be examined more directly.

### 3.3 Data sources and sample

The dataset comprised annual observations from 2012 to 2023. National-level and province-level data were drawn from the China Statistical Yearbook on Culture and Related Industries. These data included revenue and profit indicators for culture-related industries and culture and related industries, together with sector-specific revenue information for the radio and television broadcasting industry. Province-level data covered China’s 31 administrative regions. The annual structure of the dataset made it possible to compare pre-policy and post-policy trajectories around the 2017 intervention while also assessing regional variation in the magnitude and direction of change.

### 3.4 Variables and measurement

The primary dependent variables in the ITS models were total revenue and total profit in culture-related industries and in culture and related industries. These indicators were analyzed at both the national and provincial levels. In the sectoral concentration analysis, the dependent variable was per capita revenue in the radio and television broadcasting sector. The intervention variable was the 2017 digital cultural policy, coded as 0 for the years 2012–2016 and 1 for the years 2017–2023. Accordingly, 2017 was treated as the first post-policy year in the interrupted time series models, consistent with the policy’s issuance in that year and the study’s focus on policy-associated structural change from the intervention point onward.

In time-series analysis, logarithmic transformation is often used to reduce heteroscedasticity and stabilize the variance of financial indicators. However, because several provincial observations included zero or negative values, logarithmic transformation was not suitable for all series. All dependent variables used in the ITS models were therefore transformed using the inverse hyperbolic sine function (asinh). The asinh transformation approximates the natural logarithm for large values while remaining defined at zero and for negative observations, making it appropriate for skewed monetary data with non-positive values [36,37].

### 3.5 Analytical strategy

ITS designs are widely used in policy evaluation when randomized experimentation is infeasible and when a clearly dated intervention allows researchers to compare pre-intervention and post-intervention trends in the same outcome series [38,39]. ITS regression was used to estimate whether the 2017 policy was associated with changes in level and trend in the development of culture-related industries and culture and related industries. The basic linear interrupted time series model was specified as follows (Eq. 1):


$$\begin{array}{c} Y_{t}=\beta_{\text{0}}+\beta_{\text{1}}\cdot {Time}_{t}+\beta_{\text{2}}\cdot {Policy}_{t}+\beta_{\text{3}}\cdot {PostPolicyTime}_{t}+\varepsilon_{t} \end{array}$$ (1)

where *Y*_*t*_ denotes the outcome variable in year *t*, *Time*_*t*_ is a continuous variable indicating time since the start of the observation period, *Policy*_*t*_ is a binary variable c oded 0 for 2012–2016 and 1 for 2017–2023, *PostPolicyTime*_*t*_ counts elapsed time after the intervention, and *ε*_*t*_ is the error term. In this specification, *β*_1_ represents the pre-policy slope, *β*_2_ represents the immediate level change at the intervention point, and *β*_*3*_ represents the post-policy slope.

When functional-form misspecification was indicated, a quadratic ITS specification was used (Eq. 2):


$$\begin{array}{c} Y_{t}=\beta_{\text{0}}+\beta_{\text{1}}\cdot {Time}_{t}+\beta_{\text{2}}\cdot {Policy}_{t}+\beta_{\text{3}}\cdot {PostPolicyTime}_{t}+\beta_{\text{4}}\cdot \overset{\text{2}}{\underset{t}{PostPolicyTime}}+\varepsilon_{t} \end{array}$$ (2)

where *β*_*4*_ captures the quadratic post-policy slope. This specification allows the post-intervention trajectory to vary nonlinearly over time.

All ITS regressions were estimated using Newey–West standard errors to account for heteroscedasticity and autocorrelation. Model diagnostics included the Shapiro–Wilk test for residual normality, the Durbin–Watson statistic for autocorrelation, and the Ramsey RESET test for functional-form misspecification. Where the RESET test suggested misspecification, the quadratic interrupted time series model was retained.

The ITS analysis was first conducted at the national level for revenue and profit outcomes. The same framework was then applied to each of the 31 administrative regions to estimate province-specific post-policy slope coefficients (*β*_*3*_). These provincial estimates were used to assess regional heterogeneity in post-2017 development trajectories.

To quantify regional disparities in the radio and television broadcasting sector, three annual inequality indicators were calculated across provinces from 2012 to 2023: the Gini coefficient, the spatial Gini index, and the Theil index.

The Gini coefficient was used to measure overall inequality in the provincial distribution of per capita broadcasting revenue (Eq. 3):


$$\begin{array}{c} G=\frac{\overset{n}{\underset{i=1}{\sum }}\overset{n}{\underset{j=1}{\sum }}\left|yi-yj\right|}{2n^{\text{2}}\overset{―}{y}} \end{array}$$ (3)

where, *y*_*i*_ and *y*_*j*_ denote per capita broadcasting revenue in provinces *i* and *j*, *ȳ* is the mean of provincial per capita revenue, and *n* is the number of provinces.

To account for geographic contiguity, a spatial Gini index was also calculated (Eq. 4):


$$\begin{array}{c} Gs=\frac{\overset{n}{\underset{i=1}{\sum }}\overset{n}{\underset{j=1}{\sum }}\left|yi-yj\right|}{2\overset{n}{\underset{i=1}{\sum }}\overset{n}{\underset{j=1}{\sum }}\omega ij\overset{―}{y}} \end{array}$$ (4)

where *w*_*ij*_ is the spatial weight between provinces *i* and *j*, and $W\;=\;\sum_{i}\sum_{j}w_{\left\{ij\right\}}$. A binary first-order contiguity matrix was used to identify neighboring provinces.

The Theil index was used to capture entropy-based inequality and to decompose inequality into within-group and between-group components (Eq. 5):


$$\begin{array}{c} T=\frac{\text{1}}{n}\sum_{i=1}^{n}\frac{yi}{\overset{―}{y}}ln\left(\frac{yi}{\overset{―}{y}}\right) \end{array}$$ (5)

where is *y*_*i*_ provincial per capita broadcasting revenue and *ȳ* is the national mean. This decomposition made it possible to distinguish the relative contribution of internal dispersion and broader structural stratification to the rise of regional inequality over time.

For decomposition of the Theil index, provinces were grouped into digital hubs and non-hub regions. The digital hub group comprised Beijing, Shanghai, Guangdong, Zhejiang, and Jiangsu, reflecting their cumulative dominance in digital-cultural output and their central role as host locations for major national platform headquarters. All remaining provinces were classified as non-hub regions. The between-group component therefore captured inequality between these two categories, whereas the within-group component captured inequality among provinces within each category.

To assess whether the observed increase in broadcasting-sector concentration reflected discrete temporal shifts rather than a smooth monotonic trend, the Bai–Perron multiple structural break procedure was additionally applied to the annual concentration series. Specifically, structural break tests were conducted on key broadcasting-sector inequality indicators, including the Gini coefficient, the spatial Gini index, and the between-group Theil component. The Bai–Perron procedure estimates unknown break years by minimizing the residual sum of squares and comparing candidate models with different numbers of breakpoints using the Bayesian Information Criterion [40]. In this study, the Bai–Perron analysis served as a sector-specific, data-driven structural-break check for the concentration argument.

### 3.6 Robustness analysis

As a model-sensitivity check, we re-estimated the national aggregate revenue and profit series using a 2017-centered fixed-break piecewise trend model. The specification included a linear time trend, a post-2017 indicator, and a post-2017 slope-change term, enabling us to test whether the main national findings depended on the specific interrupted time-series parameterization. Outcomes were transformed using the asinh function, and coefficients were estimated with Newey–West robust standard errors. Standard diagnostic tests and information criteria were used to assess model adequacy.

### 3.7 Statistical software and data availability

All statistical analyses were conducted in R 4.3.1 (R Core Team, 2025). ITS regressions were estimated using the lmtest package (version 0.9–40) with robust standard errors from the sandwich package (version 3.1–1). Structural break tests were performed with the breakpoints function from the strucchange package (version 1.5–4). Spatial weights were constructed using the spdep package (version 1.3–13). Inequality metrics were calculated with the ineq (version 0.2–13) and reldist (version 1.7–2) packages. The dataset used in this study was made publicly available through Figshare (https://doi.org/10.6084/m9.figshare.29374145.v1).

## 4. Results

### 4.1 National-level post-policy shifts in the cultural industry

**Fig 2:**
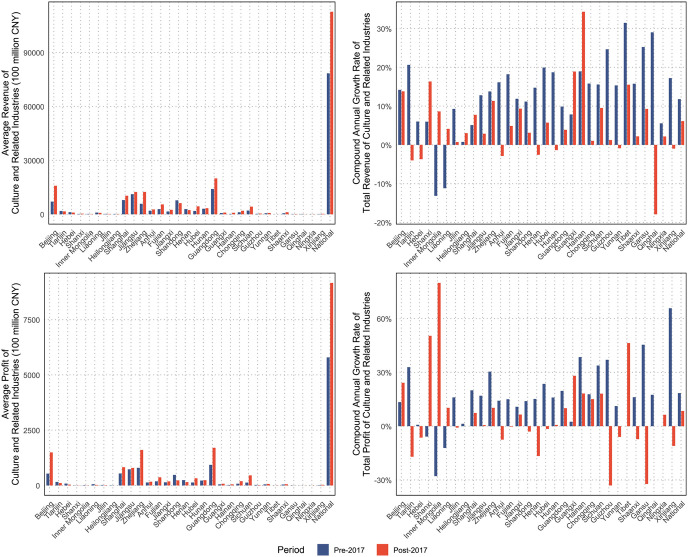
Overview of China’s culture and related industries development before and after the 2017 Opinion.

Fig 2 presents the national evolution of revenue and profit in China’s cultural and related industries before and after the 2017 policy intervention. At the descriptive level, national revenue and profit continued to increase in absolute terms after 2017, but the visual pattern indicates a clear weakening of growth momentum relative to the pre-policy period. This divergence between continuing aggregate expansion and slower post-policy growth suggests that the intervention period was associated less with an immediate collapse in national performance than with a marked change in trajectory.

The ITS estimates reported in Table 1 support this interpretation. Across all national models, the pre-policy slopes (*β*_1_) were positive and statistically significant, indicating consistent upward trajectories between 2012 and 2016. By contrast, the immediate level change at the intervention point (*β*_2_) was mostly non-significant, with notable exceptions only for culture-related service enterprises and total profit in culture and related industries. This pattern indicates that the 2017 intervention was not associated with a uniform one-year discontinuity across outcomes.

**Table 1:** Estimates and diagnostics of interrupted time series regression on the national-level revenue and profit of culture and related industries.

Variable	Pre-2017: *β*_1_	2017: *β*_2_	Post-2017: *β*_3_	Post-2017: *β*_4_	Model fit
Revenue					
Manufacturing enterprises	0.115 (0.009)***	−0.181 (0.068)*	−0.124 (0.012)***	–	all ns
Whole and retail enterprises	0.098 (0.011)***	−0.034 (0.062)	−0.085 (0.019)**	–	RESET*
Service enterprises	0.195 (0.005)***	0.135 (0.024)***	−0.064 (0.006)***	–	all ns
Total	0.130 (0.002)***	−0.077 (0.035)	−0.075 (0.005)***	–	all ns
Profit					
Manufacturing enterprises	0.115 (0.009)***	−0.102 (0.072)	−0.162 (0.015)***	–	all ns
Whole and retail enterprises	0.130 (0.026)***	0.102 (0.063)	−0.102 (0.029)**	–	all ns
Service enterprises	0.221 (0.010)***	0.093 (0.061)	−0.123 (0.018)***	–	all ns
Total	0.252 (0.054)**	0.228 (0.032)***	−0.189 (0.040)**	0.032 (0.009)*	all ns

*Note.* Newey–West standard errors are reported in parentheses. *β*_1_ denotes the pre-policy slope, *β*_2_ the immediate level change in 2017, *β*_3_ the post-2017 slope change, and *β*_4_ the quadratic post-policy slope term in the quadratic ITS specification. “Model fit” summarizes diagnostic tests for functional form and residual behavior: “all ns” indicates that the Ramsey RESET, Durbin–Watson, and Shapiro–Wilk tests were all non-significant; “RESET*” indicates evidence of functional-form misspecification in the linear model, for which the quadratic specification was retained. * *p* < .05, ** *p* < .01, *** *p* < .001.

More importantly, the post-policy slopes (*β*_3_) were negative in all eight models. For total revenue in culture and related industries, the post-policy slope was −0.075 (*p* < .001); for total profit in culture and related industries, it was −0.189 (*p* < .01). Negative post-policy slopes were also observed across all disaggregated sectors, including manufacturing, wholesale and retail, and service enterprises. Only one model, total profit in culture and related industries, retained a significant quadratic post-policy term (*β*_4_ = 0.032, *p* < .05), indicating a nonlinear post-intervention pattern in that series. Overall, the national ITS results show that the post-2017 period was associated with a broad-based deceleration or reversal in growth momentum across revenue and profit indicators, even though aggregate national totals did not immediately decline.

As a model-sensitivity check, we re-estimated the two aggregate national series using a 2017-centered fixed-break piecewise trend model. As shown in Table 2, the results were consistent across both outcomes. For total revenue, the pre-2017 trend remained strongly positive, the level shift at the 2017 breakpoint was negative but only marginally significant, and the post-2017 slope change was significantly negative. For total profit, the pre-2017 trend was likewise positive and significant, the immediate level shift at 2017 was not statistically significant, and the post-2017 slope change was again significantly negative. Overall, these estimates indicate that the principal national pattern was not a discrete one-year contraction, but a clear deceleration in growth trajectory after 2017. Model diagnostics did not indicate major specification problems, supporting the stability of this interpretation under an alternative trend-based specification.

**Table 2:** 2017-centered fixed-break piecewise trend sensitivity models.

Outcome	Pre-2017 trend	2017 level shift	Post-2017 slope change	DW *p*	BP *p*
Total revenue	0.1301***	−0.0770	−0.0746***	0.171	.130
Total profit	0.1646***	−0.0114	−0.1073***	0.075	.267

*Note.* DW = Durbin–Watson; BP = Breusch–Pagan. *** *p* <.001.

### 4.2 Provincial heterogeneity in post-policy trajectories

**Fig 3:**
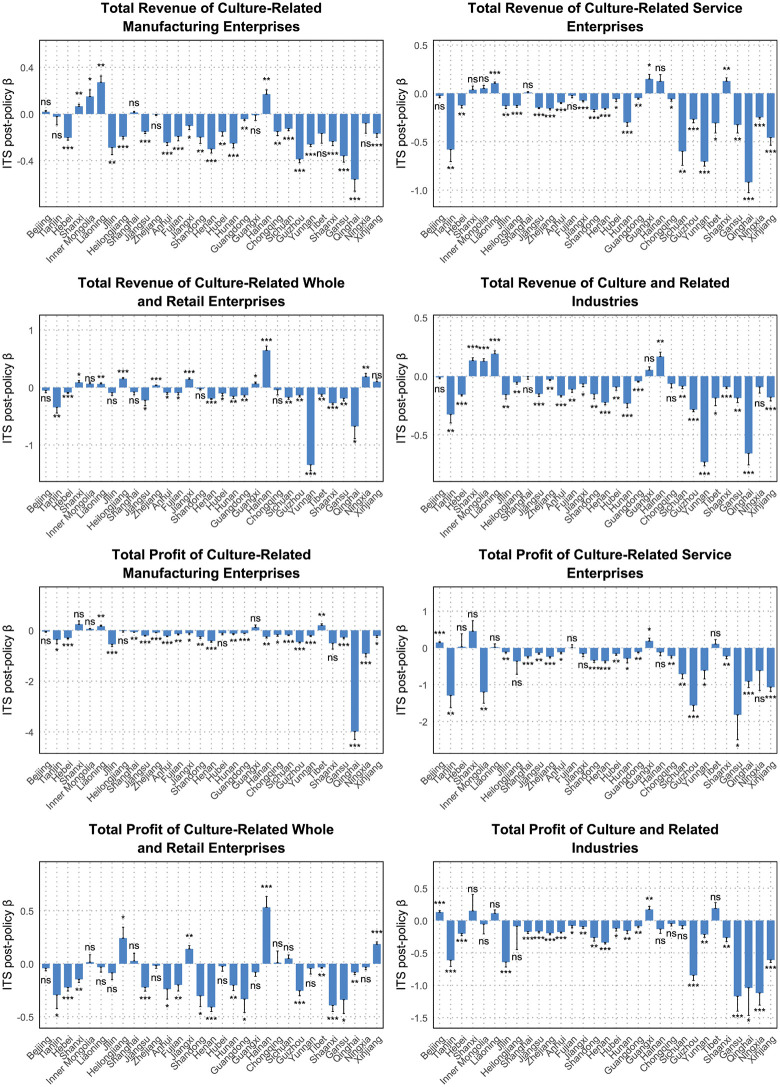
Post-policy effects on the revenue and profit of culture and related industries in 31 administrative regions. The regression coefficients are *β*_3_ in the interrupted time series regression (see text for more detail). The error bars are Newey–West standard errors. ns denotes non-significant; *denotes p < 0.05; **denotes p < 0.01; ***denotes p < 0.001.

Fig 3 summarizes the province-specific post-policy slope coefficients (*β*_3_) from the ITS models. All model diagnostics, including normality, serial correlation, and RESET results, are reported in S1–S8 Tables. These estimates show that post-2017 change was not spatially uniform. Instead, the national pattern of decelerating growth was accompanied by pronounced territorial heterogeneity in both direction and magnitude.

Across provinces, the dominant pattern was negative post-policy slopes in both revenue and profit outcomes. More than 70% of provinces showed negative *β*_3_ coefficients for total revenue, with especially steep declines in provinces such as Gansu, Qinghai, and Ningxia. Sector-specific results showed similarly widespread weakness, with negative post-policy coefficients in nearly all provinces across multiple revenue and profit categories. This pattern indicates that the aggregate post-2017 slowdown was not driven by a small number of outliers, but reflected a broad territorial weakening of growth trajectories.

At the same time, Fig 3 also indicates that the provincial effects were uneven rather than homogeneous. A limited number of provinces exhibited more resilient trajectories, while most regions experienced either statistically significant decline or non-significant post-policy stagnation. Beijing stood out as the clearest exception, retaining comparatively stronger post-policy performance than most other provinces. The provincial ITS results therefore point to widening regional heterogeneity after 2017: the post-policy period was associated not only with slower aggregate momentum, but also with a more uneven spatial distribution of growth across China’s cultural economy.

### 4.3 Structural concentration in the broadcasting sector

**Fig 4:**
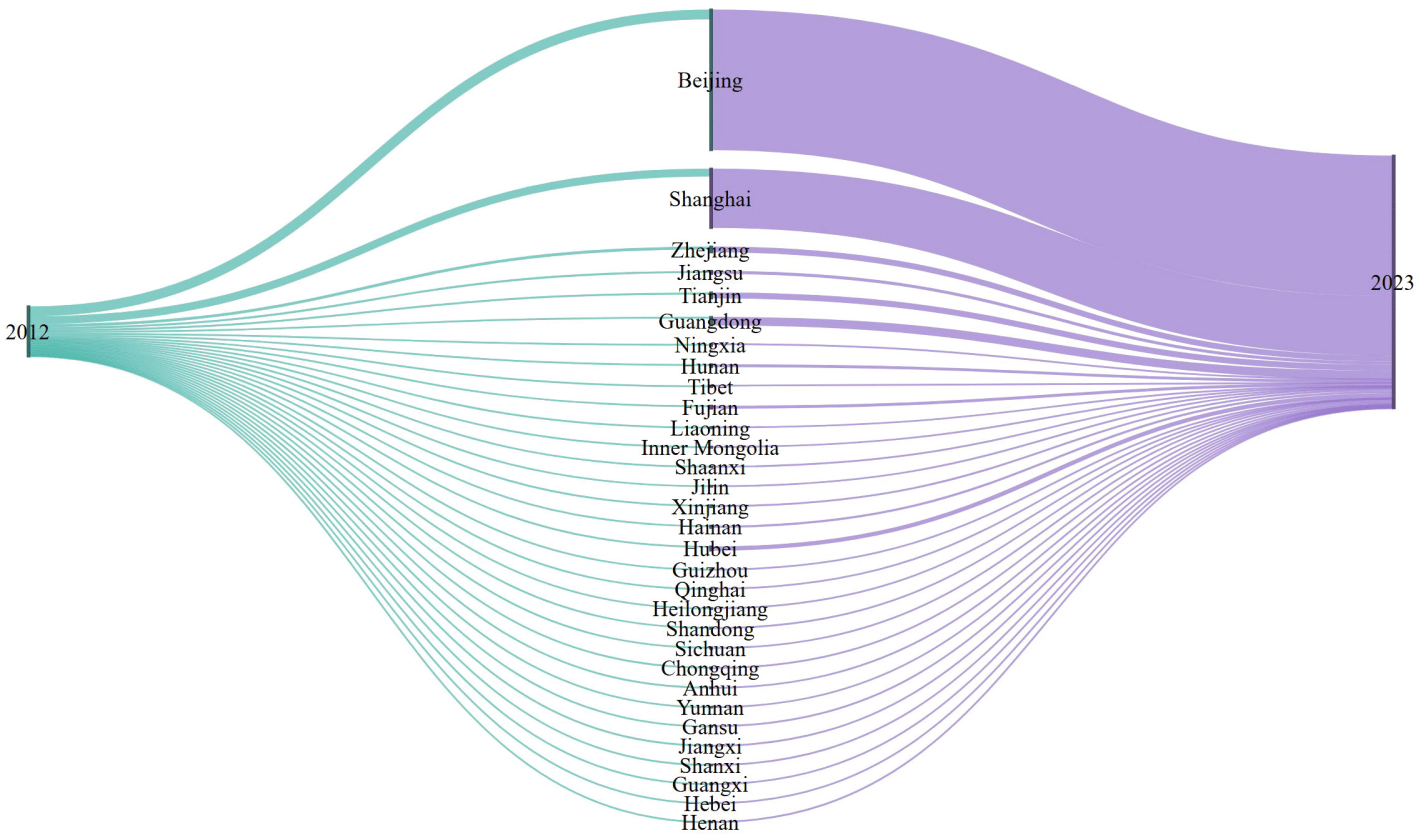
Sankey diagram of provincial per capita revenue of radio and TV broadcasting sector in 2012 and 2023.

To provide a sector-specific view of regional concentration, the analysis next examined the radio and television broadcasting sector. Fig 4 presents a Sankey diagram of provincial shares in per capita broadcasting revenue in 2012 and 2023. The visual pattern shows a clear concentration of revenue into Beijing and Shanghai over time, while most other provinces lost relative share. Even provinces that remained economically important within the broader cultural economy, such as Guangdong, Zhejiang, and Jiangsu, did not match the increasing dominance of Beijing and Shanghai in this sector.

The broadcasting sector exhibited a pronounced increase in regional concentration over the study period. As shown in Table 3, all three annual inequality indicators rose substantially between 2012 and 2023. The Gini coefficient increased from 0.442 to 0.749, the Theil index increased from 0.439 to 1.379, and the spatial Gini index increased from 0.352 to 0.642. Taken together, these trends indicate that per capita broadcasting revenue became increasingly concentrated across provinces during the post-policy period, and that this concentration was not only distributive but also geographically structured.

**Table 3:** Annual inequality measures across Chinese provinces based on per capita revenue of the radio and TV broadcasting sector.

Year	Gini coefficient	Spatial Gini index	Theil index (Total)	Theil index (Within)	Theil index (Between)
2012	0.442	0.352	0.439	0.155	0.283
2013	0.472	0.384	0.505	0.181	0.324
2014	0.536	0.417	0.632	0.196	0.436
2015	0.572	0.452	0.738	0.228	0.510
2016	0.623	0.502	0.917	0.298	0.619
2017	0.660	0.550	0.997	0.443	0.554
2018	0.717	0.599	1.226	0.543	0.683
2019	0.753	0.621	1.421	0.578	0.843
2020	0.776	0.640	1.584	0.667	0.917
2021	0.812	0.659	1.762	0.706	1.057
2022	0.834	0.684	1.862	0.667	1.194
2023	0.841	0.689	1.839	0.647	1.192

The decomposition of the Theil index further clarifies the structure of this concentration. In the earlier years of the series, between-group inequality was already the larger component, rising from 0.283 in 2012 to 0.619 in 2016, while within-group inequality remained comparatively modest, increasing only from 0.155 to 0.298 over the same period. After 2017, however, both components increased sharply. Within-group inequality rose to 0.443 in 2017 and reached 0.647 by 2023, while between-group inequality rose to 0.554 in 2017 and 1.192 by 2023. Thus, by the end of the observation period, broadcasting-sector inequality reflected both stronger divergence between digital hubs and non-hub provinces and greater internal differentiation within those groups themselves.

To assess whether this increase in concentration reflected discrete temporal shifts rather than a smooth monotonic trend, Bai–Perron multiple structural break tests were applied to the key annual concentration series. As reported in Table 4, model fit improved consistently as additional breakpoints were introduced, with the two-break specification yielding the lowest Bayesian Information Criterion values for all three principal indicators. Across the preferred specifications, 2017 emerged consistently as a structural breakpoint: both the Gini coefficient and spatial Gini index identified breaks in 2014 and 2017, while the between-group Theil component identified breaks in 2017 and 2020. This pattern suggests that broadcasting-sector concentration underwent a common structural shift around the 2017 policy intervention, followed by a later intensification of the interregional divide.

**Table 4:** Bai–Perron structural break tests for key broadcasting-sector concentration series.

Series	Model	Break years	Residual sum of squares	Bayesian Information Criterion
Gini	m = 0	None	0.218	−9.01
	m = 1	2017	0.048	−24.8
	m = 2	2014, 2017	0.0205	−32.4
Spatial Gini	m = 0	None	3.0593	22.6
	m = 1	2017	0.570	5.0
	m = 2	2015, 2018	0.2449	−2.7
Theil index (Between)	m = 0	None	0.1605	−12.7
	m = 1	2016	0.0283	−31.1
	m = 2	2014, 2017	0.0132	−37.8

*Note.* m denotes the number of structural breaks allowed in the Bai–Perron model.

Taken together, the results suggest that the post-2017 period was associated with stronger territorial concentration in broadcasting revenue, centered above all on Beijing and Shanghai and increasingly expressed through the widening gap between leading digital hubs and other provinces.

## 5. Discussion

### 5.1 Uneven post-policy development in China’s cultural economy

This study examined whether China’s 2017 digital cultural policy was associated with changes in the economic geography of the cultural industry. Three findings stand out. First, at the national level, the post-2017 period was associated with a broad weakening of growth momentum across revenue and profit indicators, even though aggregate totals did not immediately collapse. Second, the provincial ITS results showed that this deceleration was territorially uneven, with most provinces exhibiting negative post-policy slopes and only a limited number of regions demonstrating more resilient trajectories. Third, the broadcasting sector exhibited a sharp increase in regional concentration, reflected in the growth of the Gini coefficient, spatial Gini index, and Theil index, together with structural-break evidence pointing to a common shift around 2017. Taken together, these results indicate that post-policy development did not take the form of broad-based territorial diffusion. Instead, the period after the policy intervention was associated with slower aggregate momentum and a more unequal regional distribution of gains within China’s cultural economy.

These findings are important because they move the analysis of digital cultural policy beyond simple questions of aggregate expansion. A policy may coincide with continued national growth in absolute terms while still being associated with increasing territorial divergence. In this case, the combination of national deceleration, provincial heterogeneity, and broadcasting-sector concentration suggests that the relevant outcome is not whether development ceased altogether, but how it was spatially redistributed after the policy intervention. Together, these findings provide new empirical evidence that regional concentration is itself a crucial policy-relevant outcome.

### 5.2 State-amplified platform inequality as a mechanism of territorial divergence

The results are consistent with our proposed framework of state-amplified platform inequality. As defined earlier, this concept refers to a process in which state-led digital policy interacts with platform-based accumulation dynamics to intensify territorial disparities by disproportionately channeling infrastructure, institutional support, visibility, and market access toward already advantaged regions. The empirical pattern observed here fits that logic in three related ways.

First, the findings suggest that state-led digital modernization did not simply overlay a neutral developmental framework onto an otherwise open field. Rather, the policy appears to have operated in a landscape already marked by uneven institutional capacity, differential infrastructural endowments, and strong platform-centered tendencies toward cumulative advantage. This is precisely the setting in which scale, network effects, and visibility are likely to reinforce pre-existing regional asymmetries rather than overcome them [41–44]. The post-2017 weakening of aggregate growth momentum, combined with widening provincial heterogeneity, suggests that the benefits of digital cultural development were not broadly diffused across territories.

Second, the broadcasting results indicate that this territorial divergence was not confined to general economic indicators, but was expressed strongly within a strategically important cultural sector. Broadcasting is especially revealing because it lies at the intersection of state oversight, symbolic power, and scalable digital dissemination. The observed rise in both overall and spatial inequality within this sector therefore suggests that platformized cultural development became more concentrated in territorially privileged nodes, rather than more evenly distributed across provinces. In this sense, the broadcasting findings provide sector-specific evidence that the interaction between centralized policy and platform logics may deepen rather than dilute regional concentration.

Third, the results suggest that the territorial effects of digital policy are better understood as relational and cumulative than as the simple product of either market forces or administrative intervention alone. The framework proposed in this paper does not argue that concentration emerged solely because the state intended to privilege certain provinces, nor that the observed divergence was merely the spontaneous outcome of platform markets. Rather, the evidence is more consistent with a co-produced process in which policy-guided modernization and platform accumulation reinforced one another. As a result, already advantaged regions were better positioned to translate state recognition and infrastructural support into continued centrality in the cultural economy. This is the core mechanism that the concept of state-amplified platform inequality is intended to capture.

### 5.3 China in comparative perspective: platformization, state coordination, and regional inequality

The findings both align with and extend existing scholarship in three main areas. The first is the literature on platform capitalism. A growing body of research has shown that platforms concentrate power through network effects, data aggregation, infrastructural control, and scale economies, producing winner-take-most dynamics and highly uneven landscapes of value creation [45–47]. The present study is broadly consistent with that literature insofar as it documents a pattern of intensified concentration and territorial divergence. However, it also extends that literature by showing that such concentration can be reinforced by state-led modernization rather than emerging only through relatively market-centered platform competition. In this respect, the paper brings platform capitalism scholarship into closer dialogue with questions of governance structure and territorial policy.

The second relevant conversation concerns state-led digitalization and digital governance in China. Prior research has emphasized that the Chinese state is deeply involved in shaping digital development through regulation, strategic guidance, infrastructural prioritization, and institutional coordination [11,23–25]. This study is consistent with that literature in treating the state not as an external regulator but as a constitutive actor in the formation of digital markets and developmental priorities. At the same time, the present findings add an important spatial dimension. Existing work has often focused on governance, regulation, or state–platform relations at the national level. By contrast, this study shows that the consequences of state-led digitalization are also territorially uneven within the national space, and that a centrally coordinated digital policy may coincide with widening regional divergence rather than more balanced development.

The third body of scholarship concerns regional inequality and digital geography. Recent studies have examined whether digitalization mitigates or exacerbates regional disparities, often reaching mixed conclusions depending on scale, sector, and institutional setting [48–52]. The present study supports the broader claim that digital development is geographically uneven, but it goes further by locating that unevenness within a specific policy regime and by identifying a concrete sectoral site in which concentration intensified. In this sense, the paper contributes to digital geography not merely by documenting inequality, but by showing how platformized accumulation and state-led policy can become mutually reinforcing in the territorial organization of cultural industries.

Taken together, these comparisons suggest that China should not be treated as a simple outlier, nor as a straightforward replication of platform concentration dynamics found in liberal market economies. Instead, the case is analytically valuable because it reveals how digital platformization can be reshaped by centralized policy coordination, uneven local implementation, and pre-existing regional hierarchies. This hybrid configuration is what makes the observed pattern theoretically significant.

### 5.4 Conceptual, empirical, and methodological implications

The study makes a conceptual implication by advancing state-amplified platform inequality as a framework for understanding territorial divergence under state-led digital modernization. Existing explanations of platform concentration often privilege market dynamics [1,45], while discussions of developmental governance often under-specify the cumulative mechanisms through which platformized sectors reward already advantaged territories [53,54]. By linking these literatures, the present study offers a more integrated account of why digital modernization may deepen rather than reduce spatial inequality.

The study also makes an empirical implication by providing province-level evidence that the post-2017 period was associated with both weaker aggregate growth momentum and stronger territorial concentration in China’s cultural economy. Rather than inferring distributive consequences indirectly, the paper shows them through national trend shifts, province-specific post-policy trajectories, and a strategically important sectoral case. The broadcasting findings are especially significant because they reveal that concentration intensified not only in abstract aggregate terms, but in a concrete cultural field where state authority, symbolic visibility, and platformized dissemination intersect.

Finally, the study makes a methodological implication by demonstrating the value of combining ITS regression with structural-break analysis and inequality diagnostics in the study of digital policy. ITS alone can identify shifts in level and trend, but it is less informative about the distribution of gains across space. Inequality measures alone can detect concentration, but they do not identify whether broader growth trajectories changed around an intervention point. By combining these approaches, the paper offers a more robust way to study the spatial consequences of policy-driven digital transformation.

### 5.5 Scope conditions of the present study

The findings should be interpreted within clear scope conditions. First, the paper examines the economic-geographic and distributive consequences of digital cultural policy, not the full range of its cultural, social, or symbolic effects. Revenue, profit, and broadcasting-sector inequality are appropriate indicators for analyzing spatial concentration and uneven regional development, but they do not directly measure cultural participation, audience engagement, innovation diversity, representational plurality, or broader changes in cultural life. This boundary is important because it clarifies what the study can and cannot claim about the overall impact of digital cultural policy.

Second, the study is situated within one specific national policy regime. China is analytically revealing because it combines strong state coordination, rapid platformization, and pronounced regional unevenness, but the findings do not imply that all digital cultural policies in all countries will generate the same pattern. The paper instead identifies a mechanism that may be relevant in other settings where centralized digital modernization interacts with platform accumulation under unequal territorial conditions.

Third, broadcasting is used here as a strategically revealing sectoral case rather than as a proxy for the entirety of digital culture. Its institutional position makes it especially useful for observing concentration dynamics, but it remains one sector within a broader cultural economy. The fact that broadcasting exhibited intensified concentration does not by itself establish that every cultural domain followed the same trajectory in the same way.

Despite of these scope conditions, this paper offers a theory-informed analysis of how one major digital cultural policy was associated with uneven territorial outcomes in the cultural economy, and of how those outcomes can be understood through the framework of state-amplified platform inequality.

## 6. Policy implications

The findings suggest that digital cultural policy should be evaluated not only by aggregate indicators of growth, modernization, or technological upgrading, but also by distributive outcomes across regions and sectors. A policy may appear successful at the national level while simultaneously intensifying territorial inequality. For this reason, the assessment of digital cultural policy should include explicit monitoring of regional divergence, concentration trends, and sector-specific inequalities rather than assuming that aggregate expansion automatically indicates inclusive development.

The results also imply that more attention should be paid to capacity-building in lagging provinces. If digital cultural development is left to reinforce existing infrastructural and institutional advantages, already dominant regions are likely to continue consolidating their position. More balanced development would therefore require targeted support for regions with weaker digital-cultural ecosystems, including infrastructure, institutional coordination, local content production capacity, and access to distribution networks. Such measures would not eliminate regional differences, but they could reduce the extent to which policy-guided digitalization reproduces prior territorial asymmetries [55].

A third implication concerns sectoral governance. The broadcasting findings indicate that concentration can intensify sharply in sectors where state coordination, symbolic authority, and scalable dissemination intersect. This suggests the need for sector-specific oversight of concentration dynamics in broadcasting and related digital cultural services. Monitoring frameworks could include regular evaluation of regional revenue concentration, access barriers for peripheral producers, and the visibility structures through which cultural content is distributed.

Finally, digital cultural policy should be embedded more explicitly within broader regional development strategies. Culture is not only an economic sector but also part of the institutional and symbolic infrastructure through which regions participate in national development. Policies that concentrate value, visibility, and cultural production capacity in a small number of core regions may undermine longer-term goals of regional inclusion, cultural plurality, and systemic resilience. The broader implication is that digital modernization needs distributive safeguards if it is to avoid becoming territorially exclusionary.

## 7. Limitations and future research

This study has several limitations. First, the annual time series is relatively short. Although ITS analysis, structural-break tests, and robustness checks together provide a reasonable basis for identifying policy-associated shifts, the design cannot support very complex temporal modeling and cannot fully rule out all contemporaneous macro-level influences. The findings should therefore be interpreted as evidence of policy-associated structural change rather than as definitive proof of exclusive causation.

Second, the analysis relies on economic and distributive indicators. This is appropriate for the paper’s bounded focus on economic geography and regional concentration, but it does not capture broader social or cultural outcomes such as audience participation, cultural diversity, creative innovation, or symbolic inclusion. Future work should therefore extend the analysis beyond revenue and profit to examine whether state-led digital cultural policy also reshapes participation, visibility, or diversity in more substantive cultural terms.

Third, the broadcasting sector is used as a strategically revealing case, but it remains a partial one. Future research could examine whether similar concentration dynamics are evident in other digital cultural sectors, including online video, music, gaming, publishing, or livestreaming. Comparative sectoral analysis would help determine whether broadcasting is unusually concentrated because of its institutional status or whether it reflects a broader pattern across China’s digital cultural economy.

Fourth, the province-level design cannot directly identify firm-level or platform-level mechanisms. The present study shows territorial outcomes, but it cannot fully trace how specific firms, platforms, regulatory arrangements, or infrastructural decisions translated policy guidance into regional concentration. Future research using firm-level data, platform data, or mixed-method institutional analysis would be especially valuable in clarifying the mechanisms through which state-led digital policy reshapes cultural-economic geography.

Finally, the China case raises broader comparative questions that remain open. Similar dynamics may appear in other contexts where governments actively promote digitalization in already uneven territorial settings, but comparative evidence is still limited. Future research could therefore test the framework of state-amplified platform inequality in other state-led, developmental, or hybrid digital regimes.

## 8. Conclusions

This study began with a theoretical puzzle: why might state-led digital modernization intensify territorial concentration rather than diffuse opportunity? Using China’s 2017 digital cultural policy as a case, the analysis found that the post-policy period was associated with weaker aggregate growth momentum, wider provincial divergence, and stronger concentration in the broadcasting sector. These patterns are consistent with the argument that digital platformization and selective state-led modernization can interact to deepen rather than reduce regional inequality.

The broader implication is that digital cultural policy should not be understood only in terms of innovation, modernization, or national competitiveness. Under platformized conditions, state-led digital policy can also reorganize the territorial distribution of value and visibility in ways that privilege already advantaged regions. The concept of state-amplified platform inequality captures this mechanism and offers a framework for understanding how digital development, far from automatically broadening opportunity, may instead reinforce spatial asymmetry unless distributive safeguards are built into policy design, implementation, and evaluation.

## Supporting information

S1 Table ITS model fit of post-policy effect on the total revenue of culture-related manufacturing enterprises above designated size. (DOCX)

S2 Table ITS model fit of post-policy effect on the total revenue of culture-related whole and retail enterprises above designated size. (DOCX)

S3 Table ITS model fit of post-policy effect on the total revenue of culture-related service enterprises above designated size. (DOCX)

S4 Table ITS model fit of post-policy effect on the total revenue of culture and related industries above designated size. (DOCX)

S5 Table ITS model fit of post-policy effect on the total profit of culture-related manufacturing enterprises above designated size. (DOCX)

S6 Table ITS model fit of post-policy effect on the total profit of culture-related whole and retail enterprises above designated size. (DOCX)

S7 Table ITS model fit of post-policy effect on the total profit of culture-related service enterprises above designated size. (DOCX)

S8 Table ITS model fit of post-policy effect on the total profit of culture and related industries above designated size. (DOCX)
