# Supplementary material for: State-amplified platform inequality: The economic geography of digital cultural policy in China
Source: PLoS One. 2026 May 18;21(5):e0333061. doi: 10.1371/journal.pone.0333061 (PMC13183240; doi:10.1371/journal.pone.0333061)
Supplement: S1 Table — (DOCX) [file pone.0333061.s001.docx]

**S1 Table. ITS model fit of post-policy effect on the total revenue of culture-related manufacturing enterprises above designated size.**

| **Province** | **Model** | **DW** | **RESET** | **Shapiro** |
| --- | --- | --- | --- | --- |
| Beijing | LM | 0.134 | 0.214 | 0.503 |
| Tianjin | QM | 0.354 | 0.183 | 0.635 |
| Hebei | LM | 0.276 | 0.918 | 0.984 |
| Shanxi | LM | 0.059 | 0.093 | 0.582 |
| Inner Mongolia | LM | 0.071 | 0.286 | 0.850 |
| Liaoning | LM | 0.039 | 0.027 | 0.807 |
| Jilin | LM | 0.063 | 0.048 | 0.160 |
| Heilongjiang | LM | 0.009 | 0.085 | 0.662 |
| Shanghai | LM | 0.960 | 0.504 | 0.019 |
| Jiangsu | LM | 0.014 | 0.973 | 0.269 |
| Zhejiang | LM | 0.069 | 0.562 | 0.512 |
| Anhui | LM | 0.013 | 0.391 | 0.828 |
| Fujian | LM | 0.001 | 0.161 | 0.957 |
| Jiangxi | LM | 0.016 | 0.284 | 0.621 |
| Shandong | LM | 0.004 | 0.033 | 0.182 |
| Henan | LM | 0.234 | 0.846 | 0.676 |
| Hubei | LM | 0.053 | 0.224 | 0.855 |
| Hunan | LM | 0.131 | 0.057 | 0.970 |
| Guangdong | LM | 0.099 | 0.228 | 0.846 |
| Guangxi | LM | 0.281 | 0.093 | 0.031 |
| Hainan | QM | 0.369 | 0.573 | 0.454 |
| Chongqing | LM | 0.011 | 0.100 | 0.884 |
| Sichuan | LM | 0.004 | 0.831 | 0.223 |
| Guizhou | LM | 0.045 | 0.855 | 0.837 |
| Yunnan | LM | 0.566 | 0.788 | 0.254 |
| Tibet | LM | 0.002 | 0.535 | 0.959 |
| Shaanxi | LM | 0.046 | 0.470 | 0.967 |
| Gansu | LM | 0.205 | 0.548 | 0.551 |
| Qinghai | LM | 0.212 | 0.074 | 0.219 |
| Ningxia | LM | 0.265 | 0.120 | 0.836 |
| Xinjiang | LM | 0.194 | 0.963 | 0.855 |

*Note.* LM = linear model; QM = quadratic model.
