# Supplementary material for: State-amplified platform inequality: The economic geography of digital cultural policy in China
Source: PLoS One. 2026 May 18;21(5):e0333061. doi: 10.1371/journal.pone.0333061 (PMC13183240; doi:10.1371/journal.pone.0333061)
Supplement: S2 Table — (DOCX) [file pone.0333061.s002.docx]

**S2 Table. ITS model fit of post-policy effect on the total revenue of culture-related whole and retail enterprises above designated size.**

| **Province** | **Model** | **DW** | **RESET** | **Shapiro** |
| --- | --- | --- | --- | --- |
| Beijing | LM | 0.029 | 0.057 | 0.217 |
| Tianjin | LM | 0.011 | 0.158 | 0.414 |
| Hebei | LM | 0.953 | 0.423 | 0.402 |
| Shanxi | LM | 0.111 | 0.840 | 0.221 |
| Inner Mongolia | LM | 0.826 | 0.848 | 0.325 |
| Liaoning | LM | 0.452 | 0.443 | 0.109 |
| Jilin | LM | 0.146 | 0.181 | 0.560 |
| Heilongjiang | LM | 0.176 | 0.276 | 0.961 |
| Shanghai | LM | 0.014 | 0.101 | 0.091 |
| Jiangsu | LM | 0.045 | 0.033 | 0.894 |
| Zhejiang | LM | 0.631 | 0.854 | 0.086 |
| Anhui | LM | 0.133 | 0.460 | 0.837 |
| Fujian | LM | 0.071 | 0.037 | 0.358 |
| Jiangxi | LM | 0.707 | 0.934 | 0.168 |
| Shandong | LM | 0.068 | 0.134 | 0.695 |
| Henan | LM | 0.502 | 0.502 | 1.000 |
| Hubei | LM | 0.198 | 0.561 | 0.325 |
| Hunan | LM | 0.035 | 0.025 | 0.880 |
| Guangdong | LM | 0.047 | 0.322 | 0.387 |
| Guangxi | LM | 0.297 | 0.259 | 0.505 |
| Hainan | LM | 0.686 | 0.140 | 0.794 |
| Chongqing | LM | 0.435 | 0.630 | 0.055 |
| Sichuan | LM | 0.219 | 0.456 | 0.928 |
| Guizhou | LM | 0.733 | 0.248 | 0.634 |
| Yunnan | QM | 0.094 | 0.213 | 0.895 |
| Tibet | LM | 0.120 | 0.825 | 0.229 |
| Shaanxi | LM | 0.288 | 0.971 | 0.969 |
| Gansu | LM | 0.121 | 0.016 | 0.875 |
| Qinghai | LM | 0.158 | 0.073 | 0.503 |
| Ningxia | LM | 0.193 | 0.040 | 0.576 |
| Xinjiang | LM | 0.081 | 0.370 | 0.980 |

*Note.* LM = linear model; QM = quadratic model.
