# Supplementary material for: State-amplified platform inequality: The economic geography of digital cultural policy in China
Source: PLoS One. 2026 May 18;21(5):e0333061. doi: 10.1371/journal.pone.0333061 (PMC13183240; doi:10.1371/journal.pone.0333061)
Supplement: S3 Table — (DOCX) [file pone.0333061.s003.docx]

**S3 Table. ITS model fit of post-policy effect on the total revenue of culture-related service enterprises above designated size.**

| **Province** | **Model** | **DW** | **RESET** | **Shapiro** |
| --- | --- | --- | --- | --- |
| Beijing | LM | 0.561 | 0.517 | 0.234 |
| Tianjin | LM | 0.036 | 0.003 | 0.997 |
| Hebei | LM | 0.732 | 0.316 | 0.873 |
| Shanxi | LM | 0.117 | 0.310 | 0.245 |
| Inner Mongolia | LM | 0.276 | 0.160 | 0.930 |
| Liaoning | LM | 0.407 | 0.264 | 0.432 |
| Jilin | LM | 0.103 | 0.606 | 0.025 |
| Heilongjiang | LM | 0.067 | 0.696 | 0.951 |
| Shanghai | LM | 0.547 | 0.510 | 0.853 |
| Jiangsu | LM | 0.289 | 0.044 | 0.899 |
| Zhejiang | LM | 0.050 | 0.041 | 0.082 |
| Anhui | LM | 0.466 | 0.249 | 0.143 |
| Fujian | LM | 0.317 | 0.036 | 0.907 |
| Jiangxi | LM | 0.572 | 0.806 | 0.702 |
| Shandong | LM | 0.002 | 0.001 | 0.998 |
| Henan | LM | 0.642 | 0.803 | 0.514 |
| Hubei | LM | 0.452 | 0.276 | 0.888 |
| Hunan | LM | 0.202 | 0.007 | 0.457 |
| Guangdong | LM | 0.401 | 0.167 | 0.700 |
| Guangxi | LM | 0.002 | 0.019 | 0.696 |
| Hainan | LM | 0.082 | 0.746 | 0.445 |
| Chongqing | QM | 0.508 | 0.528 | 0.189 |
| Sichuan | QM | 0.544 | 0.523 | 0.217 |
| Guizhou | LM | 0.339 | 0.106 | 0.428 |
| Yunnan | QM | 0.527 | 0.100 | 0.723 |
| Tibet | LM | 0.034 | 0.139 | 0.656 |
| Shaanxi | LM | 0.701 | 0.539 | 0.999 |
| Gansu | QM | 0.498 | 0.090 | 0.309 |
| Qinghai | LM | 0.185 | 0.290 | 0.748 |
| Ningxia | LM | 0.706 | 0.811 | 0.304 |
| Xinjiang | LM | 0.132 | 0.133 | 0.549 |

*Note.* LM = linear model; QM = quadratic model.
