# Supplementary material for: State-amplified platform inequality: The economic geography of digital cultural policy in China
Source: PLoS One. 2026 May 18;21(5):e0333061. doi: 10.1371/journal.pone.0333061 (PMC13183240; doi:10.1371/journal.pone.0333061)
Supplement: S4 Table — (DOCX) [file pone.0333061.s004.docx]

**S4 Table. ITS model fit of post-policy effect on the total revenue of culture and related industries above designated size.**

| **Province** | **Model** | **DW** | **RESET** | **Shapiro** |
| --- | --- | --- | --- | --- |
| Beijing | LM | 0.362 | 0.444 | 0.753 |
| Tianjin | LM | 0.030 | 0.008 | 0.927 |
| Hebei | LM | 0.176 | 0.892 | 0.884 |
| Shanxi | LM | 0.148 | 0.293 | 0.643 |
| Inner Mongolia | LM | 0.380 | 0.056 | 0.570 |
| Liaoning | LM | 0.066 | 0.106 | 0.487 |
| Jilin | LM | 0.003 | 0.574 | 0.377 |
| Heilongjiang | LM | 0.008 | 1.000 | 0.580 |
| Shanghai | LM | 0.060 | 0.084 | 0.274 |
| Jiangsu | LM | 0.009 | 0.799 | 0.620 |
| Zhejiang | LM | 0.100 | 0.060 | 0.663 |
| Anhui | LM | 0.018 | 0.845 | 0.348 |
| Fujian | LM | 0.009 | 0.012 | 0.951 |
| Jiangxi | LM | 0.028 | 0.355 | 0.680 |
| Shandong | LM | 0.003 | 0.944 | 0.201 |
| Henan | LM | 0.344 | 0.972 | 0.984 |
| Hubei | LM | 0.227 | 0.122 | 0.079 |
| Hunan | LM | 0.129 | 0.009 | 1.000 |
| Guangdong | LM | 0.375 | 0.550 | 0.343 |
| Guangxi | LM | 0.363 | 0.093 | 0.381 |
| Hainan | LM | 0.120 | 0.829 | 0.736 |
| Chongqing | LM | 0.668 | 0.490 | 0.229 |
| Sichuan | QM | 0.313 | 0.601 | 0.072 |
| Guizhou | LM | 0.764 | 0.369 | 0.766 |
| Yunnan | QM | 0.885 | 0.949 | 0.043 |
| Tibet | LM | 0.061 | 0.227 | 0.992 |
| Shaanxi | LM | 0.407 | 0.135 | 0.754 |
| Gansu | LM | 0.096 | 0.026 | 0.327 |
| Qinghai | LM | 0.089 | 0.051 | 0.928 |
| Ningxia | LM | 0.243 | 0.063 | 0.954 |
| Xinjiang | LM | 0.585 | 0.271 | 0.777 |

*Note.* LM = linear model; QM = quadratic model.
