# Supplementary material for: State-amplified platform inequality: The economic geography of digital cultural policy in China
Source: PLoS One. 2026 May 18;21(5):e0333061. doi: 10.1371/journal.pone.0333061 (PMC13183240; doi:10.1371/journal.pone.0333061)
Supplement: S5 Table — (DOCX) [file pone.0333061.s005.docx]

**S5 Table. ITS model fit of post-policy effect on the total profit of culture-related manufacturing enterprises above designated size.**

| **Province** | **Model** | **DW** | **RESET** | **Shapiro** |
| --- | --- | --- | --- | --- |
| Beijing | LM | 0.518 | 0.614 | 0.377 |
| Tianjin | LM | 0.237 | 0.234 | 0.001 |
| Hebei | LM | 0.040 | 0.001 | 0.598 |
| Shanxi | LM | 0.017 | 0.162 | 0.623 |
| Inner Mongolia | LM | 0.757 | 0.922 | 0.277 |
| Liaoning | LM | 0.490 | 0.686 | 0.173 |
| Jilin | LM | 0.585 | 0.219 | 0.242 |
| Heilongjiang | LM | 0.345 | 0.262 | 0.103 |
| Shanghai | LM | 0.164 | 0.765 | 0.995 |
| Jiangsu | LM | 0.039 | 0.716 | 0.876 |
| Zhejiang | LM | 0.179 | 0.870 | 0.291 |
| Anhui | LM | 0.580 | 0.424 | 0.358 |
| Fujian | LM | 0.016 | 0.117 | 0.063 |
| Jiangxi | LM | 0.006 | 0.364 | 0.661 |
| Shandong | LM | 0.069 | 0.265 | 0.051 |
| Henan | LM | 0.234 | 0.066 | 0.644 |
| Hubei | LM | 0.112 | 0.209 | 0.885 |
| Hunan | LM | 0.252 | 0.193 | 0.480 |
| Guangdong | LM | 0.359 | 0.404 | 1.000 |
| Guangxi | LM | 0.100 | 0.079 | 0.546 |
| Hainan | LM | 0.956 | 0.523 | 0.260 |
| Chongqing | LM | 0.019 | 0.710 | 0.655 |
| Sichuan | LM | 0.566 | 0.115 | 0.210 |
| Guizhou | LM | 0.181 | 0.809 | 0.158 |
| Yunnan | LM | 0.534 | 0.560 | 0.322 |
| Tibet | LM | 0.010 | 0.077 | 0.480 |
| Shaanxi | LM | 0.822 | 0.582 | 0.002 |
| Gansu | LM | 0.187 | 0.855 | 0.686 |
| Qinghai | QM | 0.816 | 0.043 | 0.771 |
| Ningxia | LM | 0.966 | 0.955 | 0.595 |
| Xinjiang | LM | 0.582 | 0.440 | 0.056 |

*Note.* LM = linear model; QM = quadratic model.
