# Supplementary material for: State-amplified platform inequality: The economic geography of digital cultural policy in China
Source: PLoS One. 2026 May 18;21(5):e0333061. doi: 10.1371/journal.pone.0333061 (PMC13183240; doi:10.1371/journal.pone.0333061)
Supplement: S6 Table — (DOCX) [file pone.0333061.s006.docx]

**S6 Table. ITS model fit of post-policy effect on the total profit of culture-related whole and retail enterprises above designated size.**

| **Province** | **Model** | **DW** | **RESET** | **Shapiro** |
| --- | --- | --- | --- | --- |
| Beijing | LM | 0.669 | 0.979 | 0.856 |
| Tianjin | LM | 0.335 | 0.664 | 0.999 |
| Hebei | LM | 0.660 | 0.598 | 0.800 |
| Shanxi | LM | 0.217 | 0.204 | 0.376 |
| Inner Mongolia | LM | 0.426 | 0.389 | 0.619 |
| Liaoning | LM | 0.917 | 0.550 | 0.674 |
| Jilin | LM | 0.022 | 0.055 | 0.833 |
| Heilongjiang | LM | 0.095 | 0.707 | 0.014 |
| Shanghai | LM | 0.553 | 0.802 | 0.871 |
| Jiangsu | LM | 0.588 | 0.291 | 0.739 |
| Zhejiang | LM | 0.491 | 0.284 | 0.473 |
| Anhui | LM | 0.201 | 0.052 | 0.841 |
| Fujian | LM | 0.104 | 0.248 | 0.673 |
| Jiangxi | LM | 0.521 | 0.689 | 0.043 |
| Shandong | LM | 0.753 | 0.484 | 0.039 |
| Henan | LM | 0.177 | 0.083 | 0.490 |
| Hubei | LM | 0.166 | 0.492 | 0.833 |
| Hunan | LM | 0.035 | 0.016 | 0.999 |
| Guangdong | QM | 0.236 | 0.508 | 0.797 |
| Guangxi | LM | 0.939 | 0.921 | 0.917 |
| Hainan | LM | 0.122 | 0.872 | 0.890 |
| Chongqing | LM | 0.166 | 0.174 | 0.982 |
| Sichuan | LM | 0.542 | 0.563 | 0.204 |
| Guizhou | LM | 0.258 | 0.037 | 0.991 |
| Yunnan | LM | 0.790 | 0.581 | 0.150 |
| Tibet | LM | 0.298 | 0.303 | 0.280 |
| Shaanxi | LM | 0.442 | 0.415 | 0.651 |
| Gansu | LM | 0.168 | 0.252 | 0.014 |
| Qinghai | LM | 0.257 | 0.263 | 0.333 |
| Ningxia | QM | 0.135 | 0.147 | 0.589 |
| Xinjiang | LM | 0.654 | 0.823 | 0.050 |

*Note.* LM = linear model; QM = quadratic model.
