# Supplementary material for: State-amplified platform inequality: The economic geography of digital cultural policy in China
Source: PLoS One. 2026 May 18;21(5):e0333061. doi: 10.1371/journal.pone.0333061 (PMC13183240; doi:10.1371/journal.pone.0333061)
Supplement: S7 Table — (DOCX) [file pone.0333061.s007.docx]

**S7 Table. ITS model fit of post-policy effect on the total profit of culture-related service enterprises above designated size.**

| **Province** | **Model** | **DW** | **RESET** | **Shapiro** |
| --- | --- | --- | --- | --- |
| Beijing | LM | 0.463 | 0.252 | 0.045 |
| Tianjin | LM | 0.084 | 0.000 | 0.536 |
| Hebei | LM | 0.099 | 0.205 | 0.203 |
| Shanxi | LM | 0.033 | 0.004 | 0.441 |
| Inner Mongolia | LM | 0.410 | 0.858 | 0.061 |
| Liaoning | LM | 0.939 | 0.422 | 0.402 |
| Jilin | LM | 0.220 | 0.077 | 0.444 |
| Heilongjiang | LM | 0.085 | 0.017 | 0.072 |
| Shanghai | LM | 0.335 | 0.777 | 0.929 |
| Jiangsu | LM | 0.343 | 0.005 | 0.335 |
| Zhejiang | LM | 0.069 | 0.125 | 0.520 |
| Anhui | LM | 0.752 | 0.816 | 0.067 |
| Fujian | LM | 0.265 | 0.197 | 0.715 |
| Jiangxi | LM | 0.055 | 0.662 | 0.007 |
| Shandong | LM | 0.014 | 0.843 | 0.335 |
| Henan | LM | 0.451 | 0.866 | 0.690 |
| Hubei | LM | 0.263 | 0.309 | 0.125 |
| Hunan | LM | 0.190 | 0.102 | 0.369 |
| Guangdong | LM | 0.171 | 0.220 | 0.497 |
| Guangxi | LM | 0.073 | 0.409 | 0.373 |
| Hainan | LM | 0.093 | 0.159 | 0.002 |
| Chongqing | QM | 0.938 | 0.758 | 0.426 |
| Sichuan | QM | 0.103 | 0.800 | 0.963 |
| Guizhou | LM | 0.370 | 0.225 | 0.412 |
| Yunnan | LM | 0.696 | 0.259 | 0.006 |
| Tibet | LM | 0.252 | 0.086 | 0.204 |
| Shaanxi | LM | 0.663 | 0.193 | 0.586 |
| Gansu | QM | 0.031 | 0.010 | 0.987 |
| Qinghai | LM | 0.171 | 0.074 | 0.989 |
| Ningxia | QM | 0.190 | 0.183 | 0.368 |
| Xinjiang | LM | 0.068 | 0.225 | 0.446 |

*Note.* LM = linear model; QM = quadratic model.
