# Supplementary material for: State-amplified platform inequality: The economic geography of digital cultural policy in China
Source: PLoS One. 2026 May 18;21(5):e0333061. doi: 10.1371/journal.pone.0333061 (PMC13183240; doi:10.1371/journal.pone.0333061)
Supplement: S8 Table — (DOCX) [file pone.0333061.s008.docx]

**S8 Table. ITS model fit of post-policy effect on the total profit of culture and related industries above designated size.**

| **Province** | **Model** | **DW** | **RESET** | **Shapiro** |
| --- | --- | --- | --- | --- |
| Beijing | LM | 0.392 | 0.204 | 0.050 |
| Tianjin | LM | 0.055 | 0.212 | 0.477 |
| Hebei | LM | 0.616 | 0.190 | 0.518 |
| Shanxi | LM | 0.028 | 0.037 | 0.027 |
| Inner Mongolia | LM | 0.431 | 0.627 | 0.736 |
| Liaoning | LM | 0.749 | 0.400 | 1.000 |
| Jilin | QM | 0.527 | 0.406 | 0.960 |
| Heilongjiang | LM | 0.066 | 0.005 | 0.052 |
| Shanghai | LM | 0.478 | 0.860 | 0.511 |
| Jiangsu | LM | 0.167 | 0.157 | 0.695 |
| Zhejiang | LM | 0.056 | 0.106 | 0.452 |
| Anhui | LM | 0.724 | 0.879 | 0.536 |
| Fujian | LM | 0.128 | 0.102 | 0.457 |
| Jiangxi | LM | 0.050 | 0.503 | 0.297 |
| Shandong | LM | 0.077 | 0.105 | 0.873 |
| Henan | LM | 0.647 | 0.930 | 0.857 |
| Hubei | LM | 0.335 | 0.289 | 0.501 |
| Hunan | LM | 0.100 | 0.010 | 0.585 |
| Guangdong | LM | 0.252 | 0.140 | 0.269 |
| Guangxi | LM | 0.165 | 0.047 | 0.466 |
| Hainan | LM | 0.045 | 0.040 | 0.207 |
| Chongqing | LM | 0.012 | 0.021 | 0.444 |
| Sichuan | LM | 0.180 | 0.294 | 0.906 |
| Guizhou | LM | 0.293 | 0.172 | 0.183 |
| Yunnan | LM | 0.810 | 0.672 | 0.134 |
| Tibet | LM | 0.080 | 0.006 | 0.976 |
| Shaanxi | LM | 0.908 | 0.745 | 0.064 |
| Gansu | LM | 0.186 | 0.085 | 0.993 |
| Qinghai | LM | 0.084 | 0.119 | 0.694 |
| Ningxia | LM | 0.944 | 0.873 | 0.775 |
| Xinjiang | LM | 0.209 | 0.870 | 0.882 |

*Note.* LM = linear model; QM = quadratic model.
